# On the Triggering Mechanisms of Upward Lightning

**DOI:** 10.1038/s41598-019-46122-x

**Published:** 2019-07-03

**Authors:** Carina Schumann, Marcelo M. F. Saba, Tom A. Warner, Marco A. S. Ferro, John H. Helsdon, Ron Thomas, Richard E. Orville

**Affiliations:** 1 School of Geosciences and School of Electrical and Information Engineering, University of the Witwatersrand Johannesburg, Johannesburg, South Africa; 2 National Institute for Space Research, INPE, São José dos Campos, Brazil; 3 Department of Physics, South Dakota School of Mines and Technology, Rapid City, South Dakota USA; 4 Institute of Aeronautics and Space (IAE), Atmospheric Science Division, São José dos Campos, Brazil; 5 Department of Physics, New Mexico Tech, Socorro, New Mexico, USA; 6 Department of Atmospheric Sciences, Texas A&M University, College Station, Texas, USA

**Keywords:** Applied physics, Natural hazards

## Abstract

Upward lightning studies took place in Rapid City, South Dakota, USA and S. Paulo, Brazil during the summer thunderstorm seasons from 2011 to 2016. One of the main objectives of these campaigns was to evaluate and characterize the triggering of upward positive leaders from tall objects due to preceding nearby flash activity. 110 upward flashes were observed with a combination of high- and standard-speed video and digital still cameras, electric field meters, fast electric-field antenna systems, and for two seasons, a Lightning Mapping Array. These data were analyzed, along with correlated lightning location system data, to determine the triggering flash type responsible for the initiation of upward leaders from towers. In this paper, we describe the various processes during flash activity that can trigger upward leaders from tall objects in the USA and in Brazil. We conclude that the most effective triggering component is the propagation of the in-cloud negative leader during the continuing current that follows a positive return stroke.

## Introduction

Upward lightning is the development of a self-propagating lightning leader from a tall object that travels upward toward the overlaying electrified storm cloud. Upward lightning can initiate without any preceding lightning activity, herein classified as self-initiated upward lightning. For self-initiated upward lightning to occur, storm electrification and the resulting presence of a cloud charge region is required to generate an electric field necessary for the initiation and growth of an upward leader. The elevated vertical shape of the tall object enhances the electric field locally resulting in conditions favorable for initiation of an upward leader. However there is no proceeding nearby lightning activity prior to the initiation of the upward leader. An upward leader from a tall object can also initiate and develop in response to an electric field change created by a nearby preceding lightning flash (also known as the triggering flash). Since the upward leader is essentially triggered by the nearby lightning activity, we refer to this type of upward lightning as lightning-triggered upward lightning.

Past research efforts have sought to characterize the type of triggering flashes and electric field environment prior to and during the initiation of upward leaders from tall objects as well as characterize weather conditions favorable for upward flashes. The following is a literature review specifically relating to lightning-triggered upward lightning.

*Wang et al*.^1^ presented electric field changes for 14 upward flashes between 2005–2006 from a wind turbine and its protection tower in Japan. It was found that 4 upward flashes exhibited only a unidirectional negative field change (physics sign convention) that correlated with the initiation and development of an upward positive leader (UPL) without any preceding flash activity. They called this type of electric field change Type 1. The remaining 10 upward flashes showed an initial positive field change in response to nearby flash activity before the field change reversed polarity to negative in response to the development of an UPL. They defined this second type of electric field change behavior as Type 2.

Later, Lu *et al*.^2^ documented direct evidence that an upward lightning flash can trigger other upward lightning of opposite polarity from nearby tall structures.

*Wang and Takagi*^3^ reported on 53 winter upward flashes to a wind turbine and its protection tower in Japan between 2005 and 2010. Here, 28 upward leaders were triggered by preceding lightning activity and 25 developed upward leaders without any preceding flash activity. The upward flashes that were preceded by nearby lightning activity were defined as “other-triggered” (formerly Type 2) whereas the upward lightning that initiated from the tall objects without preceding lightning activity were defined as “self-initiated” (formerly Type 1). Negative upward flashes, where an upward propagating positive leader initiates from the tall object and effectively brings negative charge downward, was the dominate upward flash polarity.

*Zhou et al*.^4^ reported on 205 upward flashes from the Gaisberg Tower from 2005 to 2009. Only a small minority, 26/205 (13%), were triggered by preceding lightning activity. Of those 26 lightning-triggered upward lightning flashes, 15 upward positive leaders were triggered by a preceding positive cloud-to-ground (+CG) flash and 9 upward positive leaders were triggered by a preceding intracloud (IC) flash. One of the 9 upward positive leaders triggered by a preceding IC flash experienced a reversal of current (negative then positive) during the flash and was therefore a bipolar upward flash. Furthermore, one upward negative leader (UNL) was triggered by a negative cloud-to-ground (−CG) flash and one upward negative leader was triggered by an IC flash. Therefore, 24 of the 26 lightning-triggered upward lightning events (92%) began with the development of an upward positive leaders and only two (8%) began with the development of an upward negative leaders.

*Jiang et al*.^5^ reported on 4 lightning-triggered upward flashes from a 325 m tall tower in Beijing China during two different storms in 2012. In two cases, an upward positive leader was preceded by a nearby +CG flash, and the other two cases by intracloud lightning flashes.

In 2013, a comprehensive field campaign at a windfarm in Kansas, USA sought to address wind turbine lightning attachment from both downward and upward lightning^6^. Assets included a 10-station Lightning Mapping Array (LMA), 8-station slow antenna network, 2 electric field meters (EFMs), multiple high- and standard-speed video cameras, *in situ* lightning current transient measurements for all turbines, and National Lightning Detection Network (NLDN) data. *Rison et al*.^7^ reported on three observed negative upward flashes. For one case, an IC flash resulted in negative leaders passing over the windfarm and a single UPL initiated from one wind turbine coincident with the passage of the leaders. For the two other cases, +CG flashes resulted in the initiation of UPLs from multiple turbines during the same flash.

Saba *et al*.^8^ observed upward lightning cases in both Brazil and the United States and found that 61 out of 72 (84%) and 15 out of 28 (53%), respectively, were caused by a +CG flash within 1 s before and after the initiation of the upward lightning, at a distance within 80 km. Yuan *et al*.^9^ found that 14 out of 19 (75%) of upward lightning flashes from a 325-m tower in Beijing (from 2012 to 2016) were triggered by +CG lightning flashes.

Recently, Qi *et al*.^10^ reported a three-dimensional development characteristics of an upward lightning triggered by +CG flash. Wu *et al*.^11^ reported on a multiple lightning-triggered upward flash from 7 tall structures preceded by a very intense +CG flash.

The present work describes which triggering flash type is more effective and the various processes during flash activity that can trigger upward leaders from tall objects in Brazil and USA.

## Data Presentation

The data presented here were obtained during the upward lightning studies in Rapid City, South Dakota, USA and in S. Paulo, Brazil from 2012 to 2016. Description of our experimental setup is found in the section titled “Methods” below.

A timeline of events for each upward flash was created by combining data from each of the recording assets (the description of a case is presented below as an example). The high- and standard-speed optical assets provided the time of initial brightness increase associated with a triggering flash, leader spatial propagation behavior when visible below the cloud base (from here on, referred to as the *T-leader)*, and the initiation time of the upward leaders from the towers. In some cases, the exact initiation time of the upward propagating leaders was not possible due to the low luminosity of the initial developing leader and/or poor visibility due to rain or back lighting. In these cases, an initiation time was estimated based on the leader’s position and speed once its luminosity increased to detectable levels. Once upward leaders developed, the optical assets provided further information on the development and behavior of these leaders in relation to the triggering flash. The fast antenna systems provided the time of the first field change associated with triggering flashes and the electric field change polarity and magnitude preceding upward leader initiation and during the development of the upward leaders. The challenge of interpreting field change data during lightning-triggered upward flash is that once an upward leader develops, the field change is influenced by both the ongoing triggering flash and the upward leaders that are developing from the towers. The placement of fast electric-field sensors and of electric field meters (EFMs) at multiple locations at varying distances from the towers helped to differentiate the relative contribution of each. The LMA provided the time and location of triggering flash initiation and its spatial development relative to the towers. Particularly at the moment upward leaders initiated from the towers as determined by the optical assets.

Lightning Detection Network (LDN) data was analyzed comparatively to the timeline created from the other assets. LDN event timing, polarity and magnitude (i.e. estimated peak current) were compared with fast antenna data and high-speed optical observations to further characterize impulsive events that took place during triggering flashes and upward leader development. LDN location data for these events further quantified the spatial development of these flashes.

All the upward leaders from the towers in Rapid City and in S. Paulo during the summer observations developed in response to preceding lightning activity. Based on the analysis of the data, each triggering flash was assigned a flash type of either intracloud (IC) or cloud-to-ground (CG). Once a flash type was assigned, a triggering flash component responsible for the initiation of the upward propagating leaders was determined based on the spatial and temporal analysis.

In this study, we observed that the flash components that trigger the upward leader are either the propagation of a *T-leader* or the return stroke of the triggering flash. The propagation timeframe for these two possible triggering flash components (*T-leaders* or return strokes) are quite different. Leaders propagating in virgin air travel at speeds in the 10^4^–10^5^ ms^−1^ range and can cover large distances exceeding 100 km^12,13^. Therefore, this component can have a timeframe typically in the 10 s or 100 s of milliseconds lasting up to a second or more for an entire flash. On the other hand, a return stroke travels in the 10^7^–10^8^ ms^−1^ regime and is confined to the *T-leader* network formed prior to its initiation. Thus, the timeframe for a return stroke flash component is limited from tens of microseconds up to a couple of milliseconds for the case of a horizontally extensive *T-leader* network^12^. If the upward leader starts 3 milliseconds after the return stroke, the triggering component assigned is the continuing current. This threshold used is based on high speed video data of past studies on continuing current and it avoids contamination of what could just be return-stroke pulse tails^14,15^.

To summarize, for each of the 110 upward flashes recorded, a triggering flash type, and the triggering flash components were assigned based on analysis of the correlated data (from high- and standard-speed video cameras, digital still image cameras, fast electric-field sensors, electric field meters, lightning mapping array and lightning detection networks).

## Dataset and Case Study

**Figure 1:**
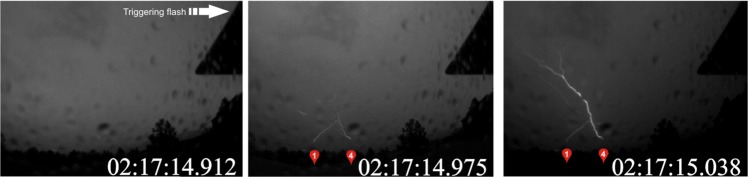
High speed video frames showing the occurrence of the triggering flash and the upward lightning flashes from Towers 1 and 4. The UTC time of each video frame (stamped at the end of the frame integration) is given as hh:mm:ss.xxx (xxx digits are milliseconds).

**Figure 2:**
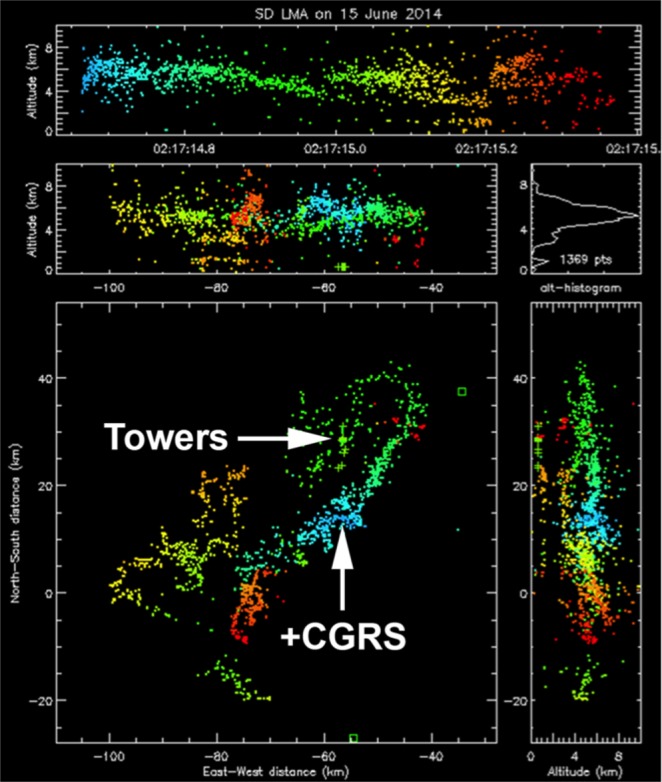
LMA source data plot of an upward flash on 15 June 2015 at 02:17:14 UT. Green + signs are tower locations.

**Figure 3:**
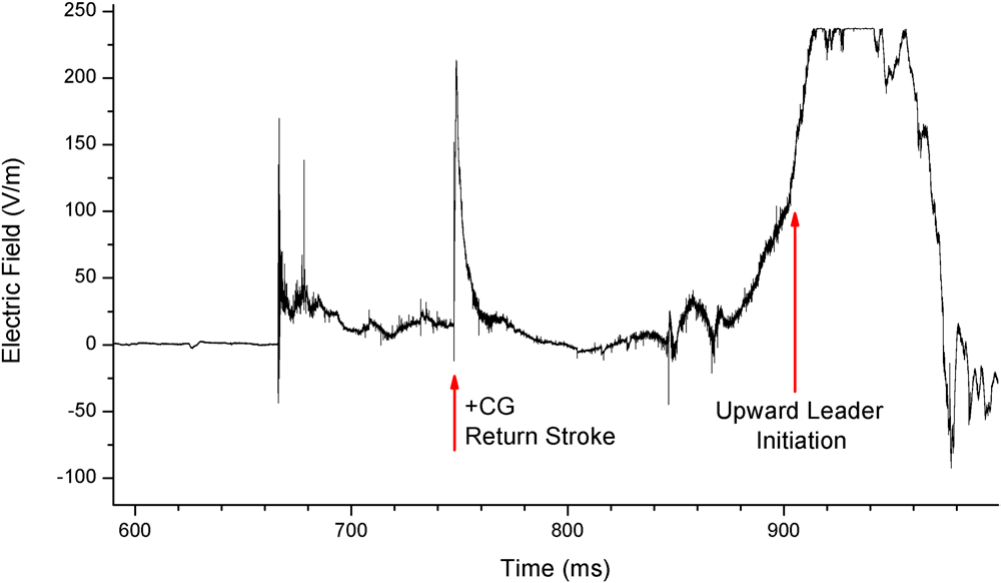
INPE fast antenna data plot from 15 June 2014 at 02:17:14 UT.

**Figure 4:**
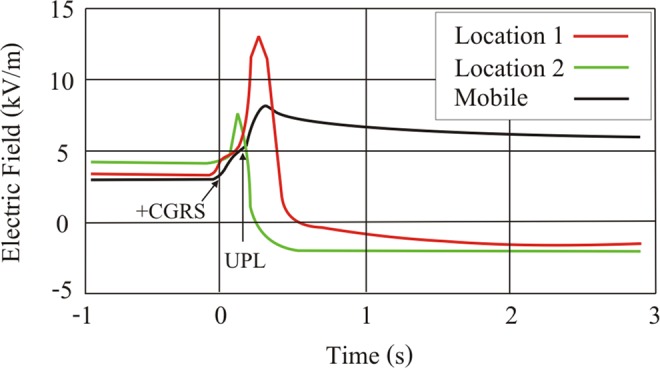
Electric field meter traces during an upward flash on 15 June 2014 at 02:17:14.747. The Mobile EFM (blue trace) was located 10 km south of Tower 4.

The following analysis of an upward lightning case will be shown to illustrate how the instrumentation described in the section “Methods” was used in this work. On 15 June 2014 at 02:17:14 UT in Rapid City, USA, upward positive leaders developed from Towers 1 and 4 (Fig. 1). Analysis of LMA data (Fig. 2) indicated that the flash initiated at an altitude of 4 km, 20 km south of Tower 4. This negative *T-leader* activity increased in altitude to 6 km while traveling northeast. At 02:17:14.747 UT, the NLDN registered a 23 kA estimated peak current +IC event 19 km south of Tower 4. A fast electric field antenna measurement (Fig. 3) and a standard-speed video showed that this event was in fact a +CG return stroke. Following the +CG return stroke, negative leader activity continued to extend northeast and traveled to a point 15 km east of Tower 4 before turning southwest and propagating near the towers at 02:17:14.900 UT. UPLs developed from Towers 1 and 4 at approximately 02:17:14.903 UT, 156 ms after the +CG return stroke. The fast antenna data showed the narrow positive field change associated with the +CG return stroke followed by a period of little field change lasting 100 ms. An increasing positive field change then began at 02:17:14.850 UT and peaked at 02:17:14.930 UT. This large field change was associated with the passage of negative leader activity near the towers as shown in the LMA data display and observed visually with the optical assets. Both the EFMs at Location 1 and Location 2 showed at positive change before reversing polarity following the development of UPLs from Towers 1 and 4. Optical assets showed that the UPL that developed from Tower 4 traveled west toward the Location 1 sensor site while the UPL from Tower 1 propagated east-northeast. The Mobile EFM located 6 km south of Tower 4 only showed a positive change associated with triggering flash activity (See Fig. 4).

For this case, negative leader activity during the continuing current of a +CG return stroke further extended the leader network that formed prior to the return stroke. The +CG return stroke failed to produce a large enough field change at the tower locations to cause the initiation of the UPLs. 156 ms following the +CG return stroke, however, the negative leader passed near the towers and caused a large positive field change at the tower locations resulting in the initiation of UPLs from Towers 1 and 4. Therefore, the triggering flash was a +CG flash and the triggering component was negative leader activity during the continuing current of a +CG return stroke.

## Results and Discussion

**Figure 5:**
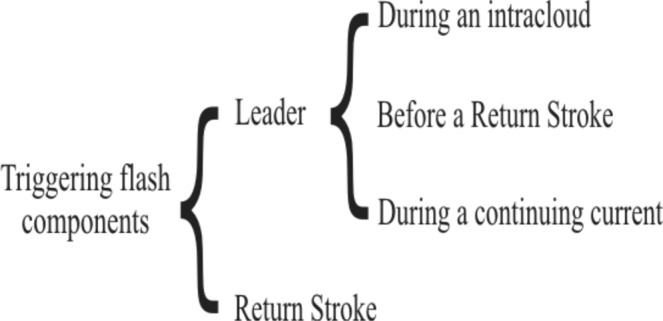
Triggering flash components that cause the upward leader initiation.

After applying a similar analysis, as mentioned above, for the whole dataset, we observed that the flash components that trigger the upward leader are either *T-leader* propagation or the return stroke. The scheme in Fig. 5 shows the triggering flash components that cause the upward leader initiation. It also shows when *T-leader* propagation triggers an upward flash (explained in the following section).

### Triggering flash component - Leader propagation

**Figure 6:**
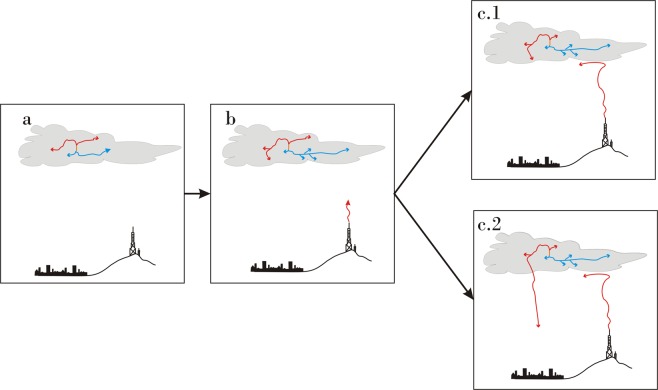
(**a**) Bidirectional intracloud leader development begins with positive (red) and negative (blue) leaders expanding from an initiation point. (**b**) The approach of negative leader initiates and upward positive leader (red) during an intracloud flash (c.1) or prior to a return stroke of a CG flash (c.2).

**Figure 7:**
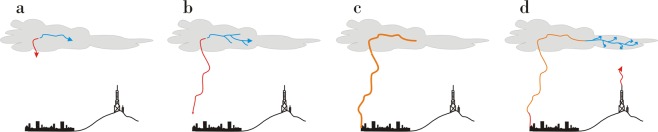
(**a**) Bidirectional intracloud leader development begins with positive (red) and negative (blue) leaders expanding from an initiation point. (**b**) The positive leader approaches the ground prior to initiating a return stroke. (**c**) The return stroke (orange) traverses the leader network. (**d**) Negative leader growth following the return stroke (blue extension beyond the return stroke channel path) passes near the tower and initiates an upward positive leader (red rising leader).

The triggering of an upward propagating leader by *T-Leader* (Figs 6 and 7) occurs in three different situations:

1. *T-Leader* during an intracloud flash (Fig. 6c.1)
2. *T-Leader* before a return stroke of a CG flash (Fig. 6c.2)
3. *T-Leader* during the occurrence of a continuing current event (Fig. 7)

**Figure 8:**
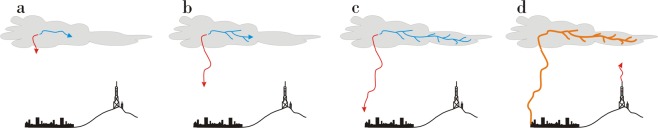
(**a**) Bidirectional intracloud leader development begins with positive (red) and negative (blue) leaders expanding from an initiation point (**b**,**c**). The positive leader approaches the ground prior to initiating a return stroke with the negative end of the leader passing near the tower (**d**). The return stroke traverses the leader network and initiates an upward positive leader from the tower (red rising leader).

When a *T-Leader* associated with an intracloud flash or *T-Leader* preceding a return stroke does not trigger an upward leader, the triggering occurs due to *T-Leader* development during the continuing current of a return stroke (Fig. 8). In this situation, an extension of the upper portions of the previously formed *T-Leader* network can occur if breakdown of virgin air continues after the return stroke reaches the outer extent of the upper portion of the *T-Leader* network^16^. In this latter case, if the return stroke channel path remains conductive and connected to ground, continuing current results and the *T-Leader* network expands further due to continued breakdown of virgin air. In the case of a +CGRS, extension will take place as continued negative breakdown of virgin air in the negative upper portion of the bipolar *T-Leader* network that formed prior to the return stroke.

### Triggering flash component - Return Stroke

When the electric field change (associated with the *T-leader* activity that propagated near the towers before the return stroke) does not reach the threshold for upward leader initiation, this threshold may be reached by the return stroke that subsequently traverses the *T-leader* network. The return stroke’s rapid electric field change serves as the triggering mechanism for upward leader initiation. (Fig. 8).

### Occurrence of triggering flash types and components

Based on the analysis of 110 cases in US and in Brazil, we observed that the majority of triggering flash types were +CG flashes (Table 1). This result is not too different from what was found in Austria although only 13% of the total number of observed upward flashes were triggered by preceding lightning activity.

**Table 1:** Number of upward flashes and triggering flash type.

	Location	Number	Triggering flash type		
			+CG	−CG	IC
This work	Rapid City	38	29 (76%)	1 (3%)	8 (21%)
	Sao Paulo	72	65 (90%)	0 (0%)	7 (10%)
Zhou *et al*.^4^	Austria	26	15 (58%)	1 (4%)	10 (38%)

Both in USA and in Brazil, most of the upward flashes were triggered by *T-leader* propagation. In almost half of the cases the *T-leader* propagation occurs during the continuing current that follows a return stroke. The percentage of triggering components in each flash type is shown in Table 2. Note that in Rapid City the number of triggering components (42) is higher than the number of upward flashes (38). This is because different components of a triggering flash can cause leader initiation in more than one tower.

**Table 2:** Triggering components occurrence in Rapid City and in Sao Paulo.

Number of Triggering Components	Rapid City	Sao Paulo
	42	72
**Leader propagation**	**69%** (**29)**	**78%** (**56)**
during an IC	19% (8)	10% (7)
before a RS	5% (2)	13% (9)
during a CC	45% (19)	55% (40)
**Return Stroke**	**31%** (**13)**	**22%** (**16)**
+CG	29% (12)	22% (16)
−CG	2% (1)	0% (0)

## Summary

Based on the analysis of 110 cases of triggered upward flashes in US and in Brazil, we observed that the majority of triggering flash types were +CG flashes and that the component of +CG flash that causes the required electric field change to initiate upward leaders from towers are either the nearby *T-leader* propagation above towers or the return stroke. The triggering of an upward propagating leader by propagating *T-leaders* occurs in three different situations: (a) during an intracloud flash; (b) before a return stroke of a CG flash, and (c) during the occurrence of a continuing current event. When the electric field change associated with the leader activity that propagated near the towers before the return stroke does not reach the threshold for upward leader initiation, a return stroke that traverses a previously formed *T-leader* may cause the triggering of an upward flash. Both in USA and in Brazil, most of the upward flashes were triggered by leader propagation. In almost half of the cases the *T-leader* propagation occurs during the continuing current that follows a return stroke.

## Methods

The instrumentation used during the upward lightning studies in Rapid City, South Dakota, USA and in S. Paulo, Brazil were composed of optical instruments, electrical field sensing and some auxiliary instrumentation described in this section.

### Optical sensing

Optical assets included high- and standard-speed video cameras along with digital still image cameras. In South Dakota, USA, up to five high-speed cameras operating between 1,000 and 35,000 images per second (ips) were used to record lightning flash activity. Cameras were grouped into two mobile platforms, which were positioned at various locations from 600 m to 9 km from the towers. The two vehicles were spatially separated so that recordings could provide some level of 3-dimensionality when comparatively analyzed. Four of the most common locations used by the two vehicles are shown in Fig. 1a. Variable locations were chosen based on storm direction of movement, storm type and expected visibility. Each vehicle had at least two high-speed cameras with one having a wide field of view, and the other having a moderate field of view on selected towers. Each vehicle also had one camera that operated in the 1,000–2,000 ips range and another in the 10,000–35,000 ips range. The two vehicles also contained standard-speed video cameras recording in standard- and high-definition that provided 60 ips continuous video at extremely wide field of views and moderate field of views on selected towers. A single digital still camera located in each vehicle captured entire flashes on single images using sequential continuous shooting at night or via infrared trigger during the day.

Three fixed optical sites located on opposite sides of Rapid City captured continuously-recorded wide field of view standard-speed video at 60 ips as well as digital still images. Distances from these sites to each of the towers ranged from 2.5 km to 11 km.

**Figure 9:**
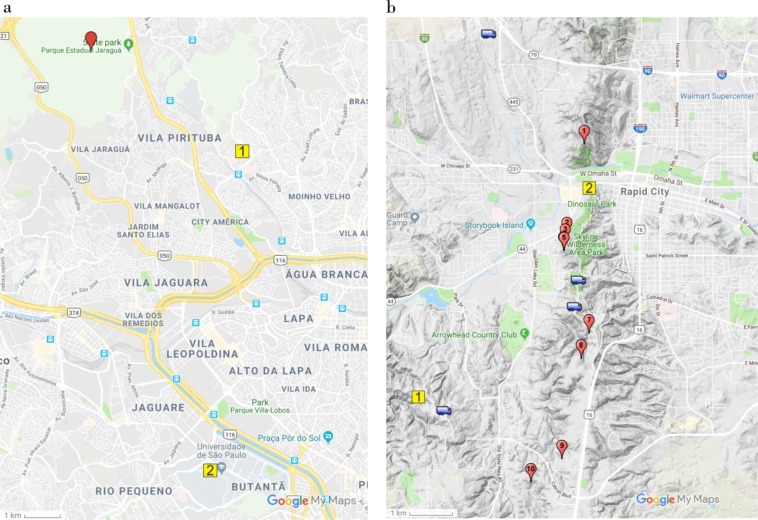
(**a**) Location of instrumentation in Rapid City (the trucks show the location of the most commonly used observation spots for the high-speed cameras; the numbered pins indicate the towers and the yellow squares show the position where the electric field antennas were installed); (**b**) Location of instrumentation in S. Paulo (the red pin indicates the tower, the yellow square 1 shows the location of the video cameras and yellow square 2 indicates where the electric field antennas were installed). Images from Map data **©**2019 Google.

In São Paulo, Brazil, optical assets included two high-speed cameras operating between 1,000 and 10,000 ips and a single digital still camera and two standard-speed video cameras recording in standard-definition that provided 60 ips continuous video at extremely wide field of views. All cameras were grouped into a platform, which was positioned at 5 km from two towers (Fig. 9b).

### Electrical field sensing

In South Dakota, USA, the National Institute for Space Research (INPE) operated two flat-plate, fast antenna systems with a sample rate of 5 million samples/second and a lower and upper frequency bandwidth of 306 Hz and 1.5 MHz respectively. A high-gain and low-gain fast antenna were placed together approximately 5 km west of the towers (Fig. 9a). At this location (yellow square 1), the dual gain system provided increased dynamic range for sensing electric field changes primarily associated with triggering flashes. A low-gain fast antenna was placed between towers 1 and 2 (Fig. 9a – yellow square 2). At this location 2, the sensor was within 1.6 km of the three towers that historically produced the most upward leaders (Towers 1, 4 and 6). This optimizes the recording of both the triggering flash field change near the tower locations and the field change resulting from the development of the upward leaders. Co-located with the fast antenna systems were electric field meters (EFMs), that sample the ambient electric field at 20 samples/sec. An additional EFM was also located on one of the mobile vehicles.

In São Paulo, Brazil, the same high-gain and low-gain fast antenna system as well as an EFM (sampling the ambient electric field at 50 samples/sec) were placed together approximately 11 km from the towers (Fig. 9b).

The field meters were factory calibrated but not calibrated to account for local field enhancement at each individual sensor site. Therefore, they were not absolutely calibrated so that electric field magnitude could only be compared relatively between the three different site locations. Data obtained from the EFMs provided insight into the initial electric field polarity as well as direction and magnitude of change during upward flashes at varying distances and directions from the towers.

The physics sign convention^12^ will be used when referring to the electric field and its change. A positive electric field is an upward pointing field when the ground is positively charged and there is negative charge overhead. A positive field change would result if there were an increase in negative charge overhead.

### Auxiliary instrumentation

For the 2014 observation season in USA, a nine station Lightning Mapping Array (LMA) provided by New Mexico Tech was installed with the centroid approximately 50 km east-southeast of the towers^17,18^.The LMA centroid was not located over the towers due to additional research objectives not related to the Upward Lightning Triggering Study (UPLIGHTS)^19^. This required that it be placed further eastward onto the high-plains. This location did, however, provide good 3-dimensional resolution of lightning generated source points for triggering and upward flashes from the towers. The LMA system recorded using 80 µs time windows^20^.

For the 2011 observation season in Brazil, a ten station Lightning Mapping Array (LMA) also provided by New Mexico Tech was installed with the centroid approximately 50 km east-southeast of the towers^21,22^.

Lightning detection network stroke data^23,24^ for a 200 km radius around the towers was obtained and analyzed for this study. Analysis provided timing and location of impulsive events detected by the networks during triggering and upward flashes in Brazil and in USA.

## Data Availability

Data for this study is available on request. Please contact the corresponding author for details.
